# Within-Trait Heterogeneity in Age Group Differences in Personality Domains and Facets: Implications for the Development and Coherence of Personality Traits

**DOI:** 10.1371/journal.pone.0119667

**Published:** 2015-03-09

**Authors:** René Mõttus, Anu Realo, Jüri Allik, Tõnu Esko, Andres Metspalu, Wendy Johnson

**Affiliations:** 1 Centre for Cognitive Ageing and Cognitive Epidemiology, Department of Psychology, University of Edinburgh, Edinburgh, United Kingdom; 2 Department of Psychology, University of Tartu, Tartu, Estonia; 3 Estonian Academy of Sciences, Tallinn, Estonia; 4 Estonian Genome Centre of University of Tartu, Tartu, Estonia; 5 Division of Endocrinology, Boston Children’s Hospital, Boston, United States of America; 6 Department of Genetics, Harvard Medical School, Boston, United States of America; 7 Broad Institute, Cambridge, United States of America; 8 Institute of Molecular and Cell Biology, University of Tartu, Tartu, Estonia; Defence Science & Technology Organisation, AUSTRALIA

## Abstract

The study investigated differences in the Five-Factor Model (FFM) domains and facets across adulthood. The main questions were whether personality scales reflected coherent units of trait development and thereby coherent personality traits more generally. These questions were addressed by testing if the components of the trait scales (items for facet scales and facets for domain scales) showed consistent age group differences. For this, measurement invariance (MI) framework was used. In a sample of 2,711 Estonians who had completed the NEO Personality Inventory 3 (NEO PI-3), more than half of the facet scales and one domain scale did not meet the criterion for weak MI (factor loading equality) across 12 age groups spanning ages from 18 to 91 years. Furthermore, none of the facet and domain scales met the criterion for strong MI (intercept equality), suggesting that items of the same facets and facets of the same domains varied in age group differences. When items were residualized for their respective facets, 46% of them had significant (p < 0.0002) residual age-correlations. When facets were residualized for their domain scores, a majority had significant (p < 0.002) residual age-correlations. For each domain, a series of latent factors were specified using random quarters of their items: scores of such latent factors varied notably (within domains) in correlations with age. We argue that manifestations of aetiologically coherent traits should show similar age group differences. Given this, the FFM domains and facets as embodied in the NEO PI-3 do not reflect aetiologically coherent traits.

## Introduction

There is a substantive literature on mean-level personality development, with numerous cross-sectional [1–5] and longitudinal [6,4,7] studies being available. Most recent of the studies have been based on the Five-Factor Model of personality (FFM) [8], which describes personality differences using five broad trait domains: Neuroticism, Extraversion, Openness (to Experience), Agreeableness, and Conscientiousness. Within this framework, older people tend to be more agreeable and conscientious and score lower on various aspects of Extraversion, Openness and Neuroticism than younger people. Despite its apparent robustness, this developmental picture may be too simplistic. A closer look at age differences within the FFM domains suggests that these five domains may not be the most optimal ways of summarizing both personality development and individual trait differences more generally.

We addressed this issue by investigating whether manifestations of the same ostensible traits show consistent age group differences. Substantial discrepancies across the manifestations in age-trajectories would suggest that their aggregation into broader traits is not necessarily warranted. Under these conditions, aggregate traits would conceal potentially valid developmental information and, more generally, would be unlikely to reflect the aetiologically consistent psychological attributes we are often hoping to study.

### Facet-Level Age Trends for the FFM Personality Traits

The 92-sample meta-analysis of longitudinal studies by Roberts and colleagues [6] showed that two aspects of Extraversion, Social Vitality and Social Dominance, showed very different mean-level changes. Social Vitality declined slightly over the life-course, while Social Dominance increased until middle-age and levelled off thereafter. Similar findings have been reported by other researchers [9,10]. Also in the Extraversion domain, Terracciano and colleagues [7] found no cross-sectional age differences in Gregariousness and Warmth across the life-span but substantial declines in the Activity, Excitement-Seeking and Positive Emotions facets. Longitudinal changes in that study were also varied across the facets of Extraversion, showing both curvilinear increases (e.g., Assertiveness) and decreases (e.g., Excitement-Seeking). Similarly inconsistent patterns were reported for a very large cross-sectional internet sample [4].

For Conscientiousness, Jackson and colleagues [11] showed in two cross-sectional samples that self-reported Industriousness and Impulse Control facets correlated positively with age, but self-reported Orderliness did not; however, there was a significant positive correlation for Orderliness in informant ratings. Using cross-sectional data, Terracciano and colleagues [7] found declines in the Competence and Achievement Striving facets and increases in the Dutifulness facet of Conscientiousness. In their longitudinal data, mean scores on the Deliberation facet increased monotonically throughout the life-course, whereas Competence and Self-discipline increased until middle age, followed by declines in later years; for other facets, age differences were smaller [7]. In a longitudinal sample of 125 women, Soto and John [10] found increases in Self-Discipline and Industriousness but no age differences in Orderliness; the same study reported significant cross-sectional age differences in Industriousness but not in Self-Discipline and Orderliness. Again, Soto and colleagues [4] made similar observations.

In the Neuroticism domain, Terracciano and colleagues [7] found that, although most of its facets showed declines with age, Vulnerability may increase, at least in late adulthood. Soto and John [10] reported that Rumination and Depression facets showed significant declines in their longitudinal sample but Anxiety and Irritability facets did not; in their cross-sectional sample, only Rumination scores declined significantly. Soto and colleagues [4] showed somewhat varying age differences for Neuroticism facets in a large cross-sectional sample. Studies have also shown variability in the magnitudes of age differences among the facets of Openness [7,9,10] and Agreeableness [7,10].

### Item-Level Age Trends

These inconsistencies within traits in age-differences may potentially extend to items within facets, though this has rarely been explored. To our knowledge, a study by Lucas and Donnellan [3] is the only study to have done so. In this study a large sample of Australians provided self-ratings using 36 adjectives scored as the FFM traits. The items of three FFM domains (Extraversion, Openness, and Agreeableness) showed distinct and often opposing age differences. There were linear increases in three aspects of Extraversion—people were less withdrawn, bashful, and shy as they aged—amidst nonlinear decreases in aspects such as Talkative, Quiet, Lively, and Enthusiastic. In the Openness domain, there appeared to be a curvilinear increase in the Philosophical item, whereas the other items displayed curvilinear decreases. In the Agreeableness domain, older people were monotonically less Harsh, less Selfish and more Sympathetic, but no substantial age differences appeared in Cold and inverse U-shaped age-trajectories were found in Kind, Warm and Cooperative. The directions of the age trends tended to be similar for Neuroticism and Conscientiousness items, but there was variability in their magnitudes.

However, these adjectives referred to abstract personality characteristics of people rather than to specific behavioural, cognitive or emotional patterns as questionnaire items often do; therefore, these results can be seen as describing age-difference in facets rather than in single personality test items. Thus, although there were indications of very nuanced patterns of age differences in different specific aspects of personality, the question of item-level variability in age-trajectories remains unanswered and, to a large degree, even unasked. Since *empirically* scale scores do not exist independently of items (although *theoretically* latent traits such as the FFM traits should exist independently of their indicators), systematic trends at the level of items should not be ignored if they appear.

### Implications for Describing Personality Change

Interpretations of mean-level age differences generally rely on the assumption that these patterns have some relevance to the ‘typical individual’ (for a critique of this assumption see [12]). Allowing this, varying mean-level age differences within domains and/or facets of personality call into question the meaningfulness of observations based on these domain and facet scales.

If some facets of an FFM domain show increases with age whereas other facets show downward trends, for example, it may not be particularly meaningful to conclude that there are no age differences in the latent trait the composite domain scores are supposed to reflect. Instead, this finding would likely imply that different facets of the purported latent trait have (partly or fully) distinct developmental mechanisms. At least, it would be premature to ignore this possibility just because the nuanced pattern of age-differences is masked in the aggregate domain scores that researchers are usually working with. The same is true for possible within-facet variability. If items of the same facets have varying age group differences, this likely points to multiple developmental mechanisms operating within the purported facets. This is consistent with the recent suggestion that the hierarchy of personality characteristics has a substantively meaningful layer of specific characteristics below facets [13].

### Implications for Personality Conceptualizations

If different indicators of a trait vary in terms of age group differences, this calls into question the theoretical rationale for considering it a coherent trait in the first place. The rationale for any personality trait rests on the idea that some latent proclivity for particular kinds of behaviours, thoughts and feelings underlies it, with facets and items representing specific instantiations of this proclivity. One fundamental characteristic of such latent proclivity should be coherence in its developmental patterns [14], as otherwise multiple underlying mechanisms would be likely. If so, multiple mechanisms may imply multiple (although possibly correlated) traits rather than a single trait. The requirement of coherence is especially relevant for the FFM personality traits that are sometimes proposed as having tractable anatomical/physiological and genomic basis and thereby distinctive aetiology [15–17]. If the FFM domains, and possibly also their facets, have multiple developmental mechanisms that operate differentially within them, as would be suggested by variable developmental patterns, we may need to look for alternative and more coherent trait conceptualizations, at least for understanding the aetiology of personality differences. This question has, for example, long fuelled debate and research in the area of cognitive abilities [18].

In particular, the presence of a strong general factor linking cognitive skills of all kinds is one of the most robust observations in psychology, and this factor is generally stronger than those linking personality facets within traits or personality test items within facets. This has led many to consider general cognitive ability, or *g* or IQ, to be of primary theoretical relevance in considering cognitive abilities [19]. Indeed, the nomological net supporting the importance of general cognitive ability in predicting important life outcomes is vast [20,21]. At the same time, it has been well documented that certain cognitive skills such as processing speed peak in early adulthood and start declining immediately, whereas other cognitive skills (e.g., verbal knowledge) peak much later in adulthood and decline more slowly [22,23]. These observations have been offered as the primary evidence against the idea of general cognitive ability as a unitary construct [24]. “Variables that correlate to indicate a factor should be related to age in a manner indicating that they represent the same function or process” [25]. Such developmental decouplings of abilities have also contributed to the development of distinct research areas investigating very specific aspects of cognitive abilities such as working memory and executive function and the field of neuropsychology that seeks to identify the particular areas of the brain involved in specific cognitive functions [18].

### Is It Just the Unique Variance That Is Inconsistent?

One plausible explanation for different developmental patterns in legitimate trait constructs is that it is only the unique variances in items/facets (i.e., variances independent of the latent trait they define) that show the different developmental patterns, whereas researchers are often interested in the common variance (i.e., in the variance of items or facets that is shared with other items/facets due to common latent traits). However, given that the latent traits cannot be defined and measured independently of the items/facets (note that trait models such as the FFM were initially derived from the co-variation patterns of items), it is difficult to specify what ‘should’ be considered the common variance in the first place. What is common variance depends on the measures and samples used to define it. When there is substantial variability among the facets/items of a FFM domain in age differences (perhaps even contrasting age-trends), conclusions regarding development of the trait (the common variance) will vary depending on which specific indicators are sampled to define it. Therefore, even if age trends in common variance (trait) and unique variances (residual variances in facets/items) can be statistically separated in any given study, studies using different samples and different specific measures of the FFM domains (i.e., sampling different trait-indicators) may still produce quite different developmental patterns. With coherently defined and measured traits this would be less likely.

### Not All Inconsistencies Are Worth Attention

Obviously, not all differences in age trajectories among the specific aspects (items, facets) of broader traits (facets, domains) are of substantive importance. Some of the variation could reflect little more than random flukes. And even if age affects all items of a facet or facets of a domain alike, there can be differences in age-trajectories due to differential loadings of the items on facets or facets on domains; the magnitudes of age differences in items or facets will have to be proportional to their loadings on the trait they define. Therefore, a formal test is needed to estimate similarities and difference among items and facets in their age-trajectories properly.

One such test is provided by multi-group confirmatory factor analyses (CFA) in the framework of what is commonly known as measurement invariance (MI) testing. This framework allows estimating the degree to which the same measurement model applies across different groups (age-levels) and testing explicitly whether age-differences in trait indicators, be they facets as indicators of domains or items as indicators of facets, are significantly different from what one would expect by chance alone or by between-indicator differences in factor loadings.

### Measurement Invariance (MI) Framework

MI refers to the situation where different indicators of a latent trait define it in the same way across groups to be compared (e.g., different age groups). Different levels of MI are established by comparing increasingly more constrained measurement models across multiple groups using CFA [26]. The first and least stringent level of MI is *configural* or *structural invariance*, which establishes overall similarity of measurement models (factor structure) across groups, without any parameter equality constraints. The analysis of configural invariance should confirm that the same indicators (items or facets) measure the latent construct(s) in all groups [27]. This level of MI is followed by weak (or *metric*) invariance, which is established if the loadings of the indicators (items or facets) on the latent factor(s) they define can be constrained equal across the groups. Weak invariance indicates that the measurement units of the latent traits are equal across the groups.

However, mean-level comparisons across different groups also assume strong (or *scalar*) invariance, which is established by constraining the intercepts as well as the loadings of the indicators to be equal across groups. Strong invariance indicates that differences in the zero-points (and given metric invariance, means) of the indicators correspond to differences in the means of their respective latent constructs. Failure to establish strong MI thus indicates that the same indicator scores correspond to different latent trait levels in different groups and that the magnitudes of the group means are not proportional to the factor loadings of the indicators (in case of measurement invariance, the magnitude of group differences in indicators should vary as a function of the their loadings on the latent trait) [28]. When it comes to age-difference, failure to establish strong MI is exactly what one would expect if indicators of the latent traits show significantly (thus over and above flukes and differential factor loadings) different age group differences, at least in terms of effect sizes and possibly also in terms of direction.

Finally, if manifest scale scores that include measurement error are to be compared across groups instead of latent factor scores that are less plagued with measurement error, *strict* MI must be established. With strict MI, strong MI is present and indicator residual variances (i.e., the unique item or facet variances that are not attributable to the latent construct) are also equal across the groups being compared [28].

### MI Framework as a Tool for Substantive Analyses

Although MI is a *technical* prerequisite for group comparisons, it is also a tool for testing *substantive* research questions. By comparing measurement models across groups we can test hypotheses about the natures of the phenomena that are purportedly embodied in the measurement models. For example, the similarity of personality test factor structures across groups may provide information about the degree to which apparent personality structure is universal [29]; such tests can be readily made using the MI framework [30]. Likewise, the framework is useful for testing for the similarity *vs* variability in the age group differences of particular traits.

### Age-Related MI in Personality Research: Inconclusive Evidence

It is only recently that studies of personality trait development have started considering the need to establish full (i.e., at least strong if latent factor scores are used) MI across age-levels [11,30–34]. A number of studies have successfully established it over relatively limited periods of life [32,35–37]. Likewise, studies over more extended periods have generally concluded that MI was present, but their tests have been carried out with fairly short scales and/or item parcels, which a priori limit the power to detect any lack of MI. For example, Zimprich and colleagues [34] showed that 24 adjectives defined the five FFM domains in the same way between ages 25 and 74. Likewise, Lucas and Donnellan [3] established MI of a 15-item FFM measure across 17 age groups spanning the whole adulthood. Allemand and colleagues [38] concluded that the 50-item shortened form of the Five-Factor Personality Inventory measured the five FFM domains invariantly from adolescence to old age. In this study, however, the 50 items were aggregated into 15 parcels, so each factor was defined by 3 to 4 aggregate indicators (parcels). By virtue of aggregation, such parcels suppress at least part of item-specific variance and possible variability in age-trends thereof. Wortmann and colleagues [33] established MI in a FFM measure based on 28 adjectives over 14 age groups; in this study too, each FFM trait was defined by 3-item parcels. Finally, Marsh and colleagues compared the scores of a 15-item FFM measure across gender and across age-levels between 15 and 99 years. Based on various types of analyses they concluded that the five FFM traits (each tapped by 3 items) could be measured invariantly over age-levels. In most of these studies the used measures had just three or four indicators (in one study, Agreeableness had 5 and Openness 7 items) and when items were parcelled to form those indicators, possible unique item-specific age-effects were suppressed from the beginning.

In contrast, some studies addressing age differences across the life-course (often with more comprehensive personality measures) have not explicitly considered the question of MI [2,3,7,10,39] or have established only weak MI [40,4,5], which does not provide information about possible item-specific age-trajectories. Insufficient consideration of MI may impair the interpretability of the findings of these studies.

The paucity of MI research is especially noteworthy when one looks for studies that both investigated age differences across large age-spans and considered lower-level facets in addition to FFM domains. This is an important gap in research, given that these are exactly the studies that, due to being comprehensive, could be most informative on personality trait development. For instance, we are not aware of any studies that have investigated age differences in the FFM domains using the currently most popular multi-trait personality instruments (e.g., the NEO-PI-R or NEO-PI-3) and tested for full (i.e., strong or strict) MI of the FFM domains or their facets across several decades. Furthermore, to our knowledge there are no studies that have tested MI for the facets of the FFM domains (using items as indicators) at any age.

### Aim of the Current Study

As was reviewed above, several recent studies have shown that there may be more to personality (trait) development than age differences in the broad FFM domains [3,10,7]. If different facets of the FFM domains or even items of the same facets show distinct age-trajectories, either in the magnitudes of differences or in their direction, this may have major implications for understanding and describing personality development and personality differences more generally. Yet formal tests of this remain rare, especially studies that address comprehensive personality measurement models (such as the FFM, for instance) and longer age-spans. The present study tested formally the degree to which facets of the FFM domains and items of the facets showed similar or contrasting age group differences in a large and demographically diverse sample of Estonians aged from 18 to 91 years. Our formal tests were based on the MI framework.

Although we refer to the group-differences as age group differences, we acknowledge from the outset that age-differences are confounded with possible cohort effects in these cross-sectional data. However, this does not have a major bearing on some of the main conclusions. If items of a facet or facets of a domain are inconsistent in cohort differences, this also poses a threat for the coherence of these traits and changes in them.

## Method

### Participants

Participants came from the Estonian Genome Centre (EGC) of the University of Tartu. The EGC was launched as an initiative of the Estonian Government in 2000 to create a database of health, genealogical, and genome data that would include 5% of Estonia’s population (for details see www.biobank.ee). The current EGC cohort includes over 51,000 people and roughly reflects the age, sex, and educational distribution of the adult Estonian population. Most of the EGC participants were randomly selected and recruited by general practitioners (GPs) and hospital physicians from among individuals visiting their offices, but volunteers were also recruited. Each participant signed an informed consent form (available at www.biobank.ee), participated in a computer-assisted personal interview at the doctor’s office, and donated a blood sample. A subset of the EGC cohort was asked to complete a self-report personality questionnaire. This subset included 600 of 1,000 randomly selected participants who had been interviewed earlier and were approached via mail with an additional request to provide personality information, and 2,175 of the most recently recruited participants who were willing to provide personality information. After excluding protocols with more than 10% missing responses in the personality questionnaire (data for 44 people, i.e., 1.6%), we used 2,711 respondents (mean age 46.04, SD = 17.33, range 18 to 91; 1,473 women). Of these, 8.1% had basic, 24.8% secondary, 27.4% vocational, and 39.7% higher education.

This research was approved by the Research Ethics Committee of the University of Tartu (approvals: 206/T-4 22.08.2011; 166/T-21 17.12.2007; 170/T-38 28.04.2008).

### Measures

*Personality*. The NEO-PI-3 [41] is a slightly modified and more readable version of the Revised NEO Personality Inventory (NEO-PI-R) [42]. Likewise, the Estonian NEO-PI-3 [43] is a modified version of the Estonian NEO-PI-R [44]. The NEO-PI-3 has 240 items that measure 30 personality facets which are grouped into the five FFM domains, such that each domain score is a composite of six facet scores. Participants respond on a 5-point Likert scale ranging from “completely disagree” to “completely agree”.

### Statistical Analyses for Facets

#### Establishing single-group measurement models for facets

For each of the 30 NEO-PI-3 facets, we first constructed single-group unidimensional CFA models that would serve as the basis for the main analyses comparing multiple age groups. For this, all items were regressed on their assigned single latent traits, which had their variances fixed at 1 to identify the models. Initially, we assumed complete local independence (i.e., residual variances were not allowed to correlate), which is a major prerequisite of latent factor models. Local independence means that items are correlated only because they are reflections of the same underlying traits scores and at any fixed level of the latent trait they become independent. However, where models applying the local independence assumption did not produce acceptable fit—defined as comparative fit index (CFI) at least. 95 or higher and root mean square error of approximation (RMSEA). 08 or lower [45]—we checked modification indices to determine the item pairs that correlated over and above being indicators of the posited latent traits.

Because these models served as the basis for all further analyses and therefore had to fit the data reasonably well, as many item pairs were allowed to have correlated residuals as was needed to obtain acceptable model fit. The residual co-variances were thus allowed on a purely statistical basis. In many cases they were conceptually obvious, (e.g., two N5: Impulsiveness items referring to over-eating had a very high residual correlation; see S2 File), but this was not always the case. Although relying only on modification indices may have risked capitalizing on chance, we judged this to be a more straightforward and less risky strategy than allowing for residual co-variances based on rational analysis of item content. Initial model fit was generally poor and residual co-variances therefore pervasive; working out all necessary residual co-variances for the 30 scales would have entailed a very large number of subjective decisions. Further, because not all necessary (by statistical criteria) residual co-variances were immediately obvious, some of them would have remained undetected and model fit thereby suboptimal. Therefore, we considered the possibility of being occasionally overly liberal in allowing items to have residual co-variances less harmful than the possibility of letting unmodeled co-variances bias model estimates elsewhere. This decision was consistent with the fact that most factor models in personality psychology, including those that gave rise to the FFM and its questionnaires in the first place, have been exploratory in nature; designing well-fitting measurement models and considering residual co-variances have therefore not been priorities in designing many personality measures including the most popular ones such as the NEO-PI-3 used in this study.

Of course, allowing for residual correlations meant admitting that latent variable models failed from the outset but we followed the common practice in personality research of ignoring the substantive meaning of this failure; poorly fitting CFA models are often disregarded in practice [46,47]. We accept that this reflects the current state of personality conceptualizations, but we provide one possible explanation for this in the Discussion.

#### Testing for MI in the facets

The sample was then divided into 12 age groups consisting of between 150 and 310 people. Generally, each group spanned a five-year range but the youngest and oldest groups had larger age ranges; the proportions of men and women were not systematically different across age groups (see Table 1). The CFA models developed in the previous step were fitted in a series of multi-group CFAs. Models were identified by fixing the variance of latent variables to 1 as this would allow the loadings of all items to be estimated and compared across groups as well as provide a consistent, standard score metric for mean-level comparisons across groups. First, no parameter constraints were imposed except for fixing factor means to 0 in all groups (configural MI). Then we introduced factor-loading equality constraints across groups with factor means still set to zero in all groups (weak MI), then released factor means of groups except for the youngest age group but constrained item intercepts as well as loadings equal across groups (strong MI). For completeness, we also tested for strict MI by further constraining the item residuals equal in all groups.

**Table 1:** The number of people in each age group.

Age group (age-range)	N	Men (%)
18–24	310	41.0
25–29	299	47.5
30–34	253	52.2
35–39	269	45.5
40–44	254	47.6
45–49	208	47.1
50–54	211	36.5
55–59	191	45.0
60–64	188	38.8
65–69	193	51.3
70–74	185	45.9
75–91	150	50.7

Each form of MI (e.g, strong) was considered to hold when the decline in CFI (ΔCFI) of the respective model (e.g., the strong MI with intercept equality constraints) compared to the previous model (e.g., the weak MI model with only loading equality constraints) was not greater than. 01 [48, 49]. We also report whether the 90% confidence intervals of the RMSEAs of the respective models overlapped, as their non-overlap has also been suggested as a criterion for failure of MI [38]. However, this criterion (RMSEACI) is more liberal than the ΔCFI as RMSEA penalizes model complexity, so increasingly more constrained models (which have greater degrees of freedom and are therefore simpler) have a ‘head start’ that may mask group differences in measurement model parameters.

We planned to set item residual co-variances equal in all groups at all levels of MI testing, so as not to confound this source of mis-specification with mis-specifications resulting from factor loadings, intercept, and residual variances. Before doing this, however, it had to be established that this did not impair model fit. For this, configural MI models with residual co-variances constrained equal across age groups could not fit data worse than those without the residual co-variances equality constraints, according to the ΔCFI and RMSEACI criteria.

#### Establishing partial MI

Next, steps were taken to allow for partial MI [50]. Partial MI holds when some measurement model parameters are allowed to vary among groups. Researchers sometimes maintain that establishing partial MI allows valid cross-group comparisons of the invariant portions of the measurement models [45,51,52], although this changes the definition of the latent trait from that intended by the theory underlying the full model by removing (differentially across groups) the variance associated with whatever constraints are released. The variance removed from the definition of latent trait vanishes into the void (or, technically speaking, shows up in residual variance). As a result, establishing partial MI may help to mask the veridical complexities pertaining to age differences in personality. Here, however, we sought to establish partial MI in order to estimate the degree to which differences between fully and partially invariant measurement models could influence the conclusions regarding age-differences in the latent traits.

To establish partial MI, when weak MI did not hold based on the ΔCFI criterion, as many factor loading were released as necessary to maintain model fit. This was a step-wise procedure, as only one constraint was relaxed at a time, followed by re-fitting the model and relaxing further constraints if necessary. Factor loadings to be released were identified based on the highest average modification index for each respective parameter across all groups. That is, for each parameter, modification indices were averaged across age groups and the parameter with the highest average modification index was relaxed. When strong MI did not hold, the same procedure was followed with intercepts: the item whose intercept yielded the highest average modification index across age groups was the first to have its intercept equality constraint released, with the procedure repeated until partial MI was established.

Where an equality constraint was released, it was simultaneously done in all age groups. This, along with the procedure of choosing constraints to be released based on *average* modification indices across groups, was done to decrease the chances of capitalizing on chance and to allow for systematic age-trends in model parameters (e.g., more or less linear age group differences in intercepts). First, making relaxing decisions based on, and relaxing parameters in, single age groups (e.g., a factor loading for a particular item in the age group of 40 to 44 years) would have entailed the possibility of tweaking thousands of parameters (e.g., for intercepts only, 30 facets * 12 age groups * 8 items = 2880 parameters) and thereby increased chances of capitalizing on chance. Second, we expected group differences in model parameters to be systematic rather than specific to single age groups. This was especially true for intercepts, given the above-reviewed evidence that items of the same scales often show different developmental trajectories. That is, it was considered possible that for a given level of a latent trait, an intercept would increase or decrease relatively linearly across age groups rather than show a decrease or increase in only one or a few age groups.

#### Variable and estimator specification

Single test items were specified as continuous variables. Ideally, it would have been appropriate to treat items as ordinal (ordered-categorical) variables but this was complicated by the fact that many NEO-PI-3 items had very skewed distributions with only very small numbers of people choosing extreme response categories (for example, in 33 of the 240 self-report items one of the five response categories was chosen by less than 1% of respondents; in 13 items, the least popular category was endorsed by less than 0.5% of respondents) and therefore splitting the sample into age groups resulted in several items having zero frequencies for some response categories in some age groups (even if we had used just as few as, say, four age groups). Given that for categorical variables each category threshold is modelled individually, this made estimating multi-group models impossible. The Robust Maximum Likelihood (MLR) estimator and scaled test statistics [53] were used, which were presumably less sensitive to non-normality of the variable distributions [54]. Since the mean of the youngest age group was set to zero and latent trait variances were set to one, the mean-score differences were effectively in standard score units based on the youngest age group. Facet scores of the age groups were compared based on the (partial) strong MI models.

### Statistical Analyses for FFM Domains

In order to estimate MI at the level of the FFM domains, hierarchical CFA models were planned, whereby items would define facets and facets would define higher-order FFM factors. However, the construction of well-fitting hierarchical models proved very difficult already in the single-group analyses (tens or hundreds of residual correlations and secondary loadings were needed to obtain acceptable models even when more liberal criteria for model fit such as CFI ≥. 90 were used). We therefore used factor scores (FS) of facets as indicators of the latent FFM domains. This was intended to remove the item-specific variance that was likely the main source of mis-specification (although it was our focus in the item-level analyses) and it allowed for simpler models with much fewer parameters. Here and henceforth, the FSs were obtained from ordinary least squares factor analyses (a single factor was extracted from the 8 items of each scale). Otherwise, baseline model construction, MI testing and partial MI-establishing procedures were similar to those described above for facets.

### Software

All analyses were carried out in the R statistical environment [55]; CFA models were fit using the ‘lavaan’ package [56]; exploratory factor analyses was done with the ‘psych’ package [57]; and CFA diagrams were drawn using the ‘semPlot’ package [58]. Covariance matrices for all age groups are given in S1 File.

## Results

### Establishing Single-Group Measurement Models for Facets

Most of the facet-level models based on the full sample did not fit data acceptably and up to five item residual variance correlations had be allowed. The fit indices of the models before and after allowing for residual variance correlations are given in Table 2; for reference, the models with standardized loadings and residual correlations are graphically shown in S2 File. The facet whose theoretical structure matched empirical data without residual variance correlations worst was N5: Impulsiveness (CFI = .56, RMSEA = .152), with the biggest issue being a very high correlation between two items that referred to eating too much; the residual correlation between these two items was higher than any item’s loading on its facet (r = .54, see S2 File). This facet illustrates one possible reason for residual correlations: many items have very similar specific content and thereby correlated over and above their associations with the latent trait they are assumed to define. Alternatively, for example, the residual correlations may point to wrong trait-conceptualization in the first place [59].

**Table 2:** Fit statistics for the facet models.

	No residual variance correlations			With residual variance correlations			
	χ2	CFI	RMSEA [90% CI]	χ2	df	CFI	RMSEA [90% CI]
N1: Anxiety	350.36	.923	.078 [.072;. 085]	141.86	18	.971	.050 [.044;. 058]
N2: Hostility	261.21	.933	.067 [.060;. 073]	138.36	19	.967	.048 [.041;. 055]
N3: Depression	303.95	.918	.072 [.066;. 079]	119.79	18	.971	.046 [.039;. 053]
N4: Self-Consciousness	363.33	.876	.080 [.073;. 086]	144.18	17	.954	.053 [.045;. 060]
N5: Impulsiveness	1264.96	.555	.152 [.144;. 159]	127.60	17	.960	.049 [.042;. 057]
N6: Vulnerability to Stress	316.97	.911	.074 [.068;. 080]	169.04	17	.955	.057 [.051;. 064]
E1: Warmth	262.39	.916	.067 [.060;. 074]	106.13	19	.970	.041 [.034;. 048]
E2: Gregariousness	634.94	.868	.107 [.100;. 113]	175.10	19	.966	.055 [.048;. 062]
E3: Assertiveness	223. 86	.954	.061 [.055;. 068]	223.86	20	.954	.061 [.055;. 068]
E4: Activity	797.37	.841	.120 [.113;. 126]	222.55	16	.958	.069 [.062;. 077]
E5: Excitement Seeking	230.43	.946	.062 [.056;. 069]	156.07	19	.965	.052 [.045;. 059]
E6: Positive Emotion	556.96	.884	.100 [.093;. 106]	184.46	18	.964	.058 [.052;. 065]
O1: Openness to Fantasy	446.94	.912	.089 [.082;. 095]	229.01	18	.956	.066 [.059;. 073]
O2: Openness to Aesthetics	1203.94	.786	.148 [.141;. 154]	241.88	18	.959	.068 [.061;. 075]
O3: Openness to Feelings	201.17	.923	.058 [.051;. 064]	113.45	18	.960	.044 [.037;. 051]
O4: Openness to Actions	408.77	.845	.085 [.078;. 092]	97.24	17	.968	.042 [.034;. 050]
O5: Openness to Ideas	947.89	.802	.131 [.125;. 137]	245.91	18	.951	.068 [.062;. 075]
O6: Openness to Values	150.88	.909	.049 [.042;. 056]	87.95	18	.952	.038 [.031;. 045]
A1: Trust	560.40	.858	.100 [.094;. 106]	162.85	18	.962	.055 [.048;. 062]
A2: Straightforwardness	261.04	.942	.067 [.060;. 073]	212.81	19	.953	.061 [.055;. 068]
A3: Altruism	421.40	.813	.086 [.080;. 093]	123.80	18	.951	.047 [.040;. 054]
A4: Compliance	155.58	.921	.050 [.043;. 057]	91.54	17	.957	.040 [.033;. 048]
A5: Modesty	761.23	.829	.117 [.111;. 123]	187.41	15	.960	.065 [.058;. 073]
A6: Tendermindedness	531.92	.739	.097 [.090;. 104]	84.18	19	.967	.036 [.029;. 043]
C1: Competence	189.30	.924	.056 [.049;. 063]	115.35	18	.956	.045 [.038;. 052]
C2: Order	629.99	.841	.106 [.100;. 113]	203.03	18	.952	.062 [.055;. 069]
C3: Dutifulness	148.74	.933	.049 [.042;. 056]	113.23	19	.951	.043 [.036;. 050]
C4: Achievement Striving	603.87	.841	.104 [.097;. 111]	154.40	18	.963	.053 [.046;. 060]
C5: Self-Discipline	502.69	.860	.094 [.088;. 101]	170.23	17	.955	.058 [.051;. 065]
C6: Deliberation	341.56	.904	.077 [.070;. 084]	175.79	18	.953	.057 [.050;. 064]

NOTE: χ2 = Chi-square; df = degrees of freedom; CFI = Comparative Fit Index; RMSEA = Root Mean Square Error Approximation; CI = confidence intervals. * Degrees of freedom = 20. The number of the pair of residual variance correlations equals 20 minus df (column 6). Chi-square statistics were significant at p <. 001 in all cases.

Most of the models were reasonable in that items had positive and in most cases sizeable loadings on their latent traits (see S2 File). The only facet that caused concerns at this stage, even after allowing for residual correlations, was O6: Openness to Values: three of its eight items had low loadings on the latent facet and one was negative (-.10). Generally, thus, it appeared feasible to fit these models as multi-group CFA models across the 12 age groups. The fit indices of all single-group and multi-group models are given in S3 File.

### Testing for MI in the Facets

#### Calibrating the MI criteria

Before testing for age-related MI, however, we ran a pseudo-invariance test to make sure that our criterion for MI (ΔCFI) was not too strict, given the number and sizes of the age groups. To this end, we randomly shuffled participants ages, thereby forming pseudo age groups of exactly the same sizes as the real age groups, and ran all MI analyses using the pseudo age groupings. Because of the random groupings, perfect MI was expected. For 12 of the 30 facets, CFIs dropped more than. 01 (for two facets the drop was greater than. 02, with the largest drop being. 027) when the configural MI model (multi-group model with no parameter equality constraints) was compared to the single-group model. However, none of the facets failed the weak, strong or strict invariance test according to the ΔCFI criterion. For the more liberal RMSEACI criterion, no facet failed any MI test across the pseudo-age groups. From this, we concluded that the ΔCFI criterion (ΔCFI ≤. 01) was adequate for weak, strong and strict invariance testing (as there appeared no type 1 errors in the pseudo-age group-based test), but it could be too restrictive for configural MI testing, for which ΔCFI greater than. 03 seemed a more appropriate criterion of the lack of MI. Accordingly, we adopted the ΔCFI ≤. 03 criterion for configural MI and the ΔCFI ≤. 01 criterion for other forms of MI. Therefore, we had empirically calibrated and apparently fair criteria for testing for MI.

#### Equality of residual co-variances

Item residual co-variances could be constrained equal across groups as this did not entail poorer model fit for any facet, according to the ΔCFI and RMSEACI criteria. This equality constraint was therefore retained throughout further analyses. Age group differences in residual co-variances were therefore non-existent and could not confound other parameters of interest.

#### Configural and weak MI

**Fig 1:**
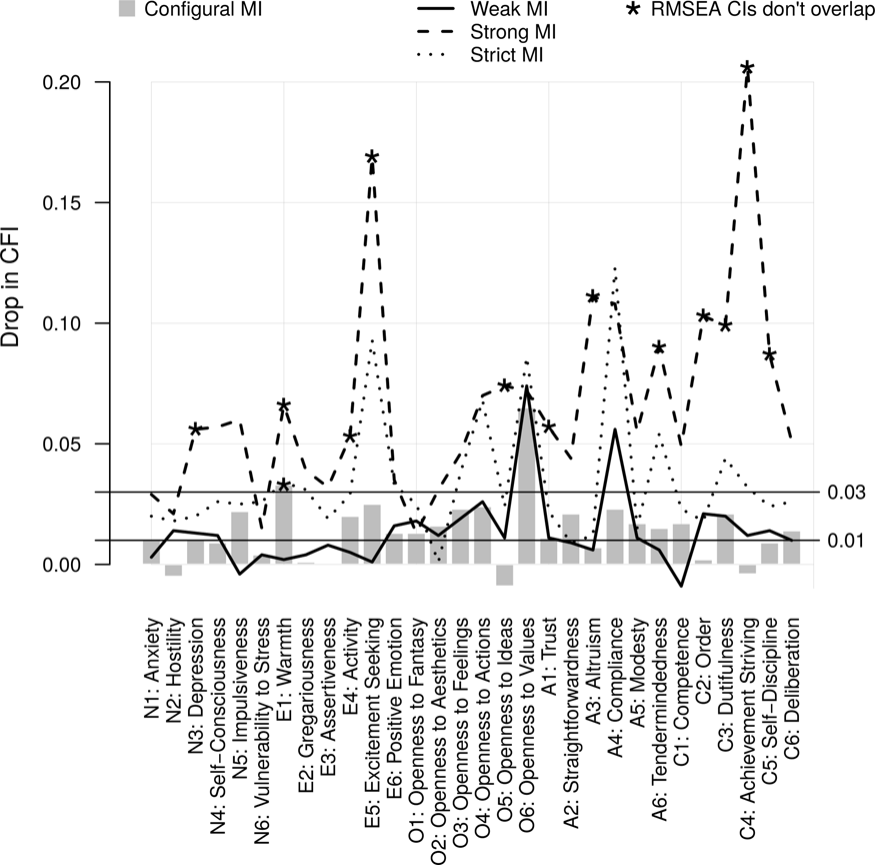
Age-related measurement invariance in the NEO-PI-3 facets: the changes in Comparative Fit Indices and Root Mean Square Error of Approximation values as a result of imposing increasingly strict parameter constraints in the measurement models of different age groups.

Two facets—E1: Warmth and O6: Openness to Values—failed the configural MI test (ΔCFI >. 03); the E1:Warmth also failed according to the RMSEACI criterion (see Fig. 1). Next, 17 of the 30 facets failed the weak MI test according to the ΔCFI criterion, with the largest ΔCFIs for O4: Openness to Actions, O6: Openness to Values and A4: Compliance. Therefore, although none of the facets failed the weak MI test according to the more liberal RMSEACI criterion, more than half of the facets appeared to have items that contributed to their latent factors with somewhat different strength at different age-levels according to ΔCFI. Although the O6: Openness to Values accumulated measurement issues at both levels of MI testing, the situation was slightly different with E1: Warmth: although the single-dimension CFA somewhat poorly fitted across the 12 age groups in the first place, the factor loadings were similar in all age groups. Taken together, there was evidence that many facets were defined differently by their items at different age levels. This needed to be dealt with by seeking partial MI (below).

#### Strong and strict MI

Pertaining to the main focus of the study, all 30 facets failed the strong MI test based on the ΔCFI criterion; in 19 facets the ΔCFIs exceeded. 05 and in five facets (E5: Excitement-Seeking, A3: Altruism, A4: Compliance, C2: Order and C4: Achievement Striving) they were greater than. 10 (see Fig. 1). For 12 facets, the RMSEA confidence intervals of weak and strong MI models also did not overlap. These results clearly pointed to variability in age differences among the items of the same facets, suggesting that the group-differences in latent facet scores were not good representations of age group differences in the behaviours, thoughts and feelings that the latent facets are assumed to cause. Quite remarkably, this was evident for all facets.

Based on the ΔCFI criterion, 28 of the 30 also failed to achieve strict MI, although no facet failed according to the RMSEACI criterion. However, since we planned to carry out all age group comparisons using latent variables, the lack of strict MI had no bearing on further analyses. It would be relevant, however, in using scale scores, as it indicates group differences in scale reliability.

#### Reliabilities of latent facets

We calculated the reliabilities of the latent facets in age groups based on configural MI models (S3 File). It appeared that reliabilities were generally lower in older age groups. Age group differences in reliabilities were consistent with the lack of strict MI.

#### Robustness analysis: Excluding the oldest age group

One may hypothesize that the oldest age group (75 and older) was the biggest source of non-invariance However, the general conclusion would have been relatively similar if this age group had been removed. For example, based on the ΔCFI criterion, 12 facets would have failed the weak MI test, whereas all facets but N6: Vulnerability would have failed to meet the ΔCFI criterion for strong and 27 facets for strict MI.

### Establishing Partial MI in Facets

#### No partial configural MI

There is no simple way of establishing partial MI for scales that fail the configural invariance test, such as E1: Warmth and O6: Openness to Values in our study. One could, of course, omit some items from the scales but such *a posteriori* ‘tweaking’ could substantially change the content of scales. For that reason, we went on with the scales as if they had met the configural MI criteria, bearing in mind that their fundamental measurement model problems may pose difficulties for the interpretability of further results.

#### Partial weak MI

In the 17 facets that failed to achieve weak MI according to the ΔCFI criterion, we identified the item that contributed the most to the lack of MI, removed its equality constraint and re-fitted the model. This procedure was repeated until the facet achieved partial weak MI. Generally one or two, but sometimes three (C2: Order), four (O6: Openness to Values) or five (A4: Compliance) equality constraints on loadings had to be relaxed (overall, loading constraints were relaxed for 30 or the 240, or 12.5%, of items).

#### Partial strong MI

In all facets that failed to achieve strong MI, partial weak MI was established as above, and then the item contributing the most to the lack of strong MI was identified and its intercept constraint was relaxed. This procedure was repeated until partial strong MI could be established. From one to six items (median across the 30 facets was three and the total count 101, i.e., 42.1% of items) had to have their intercept equality constraints removed.

We did not seek to establish partial strict MI because we based our age comparisons on latent trait scores.

### Did Establishing Partial MI in Facets Have Implications for Mean Comparisons?

#### Age group differences before and after establishing partial MI

**Fig 2:**
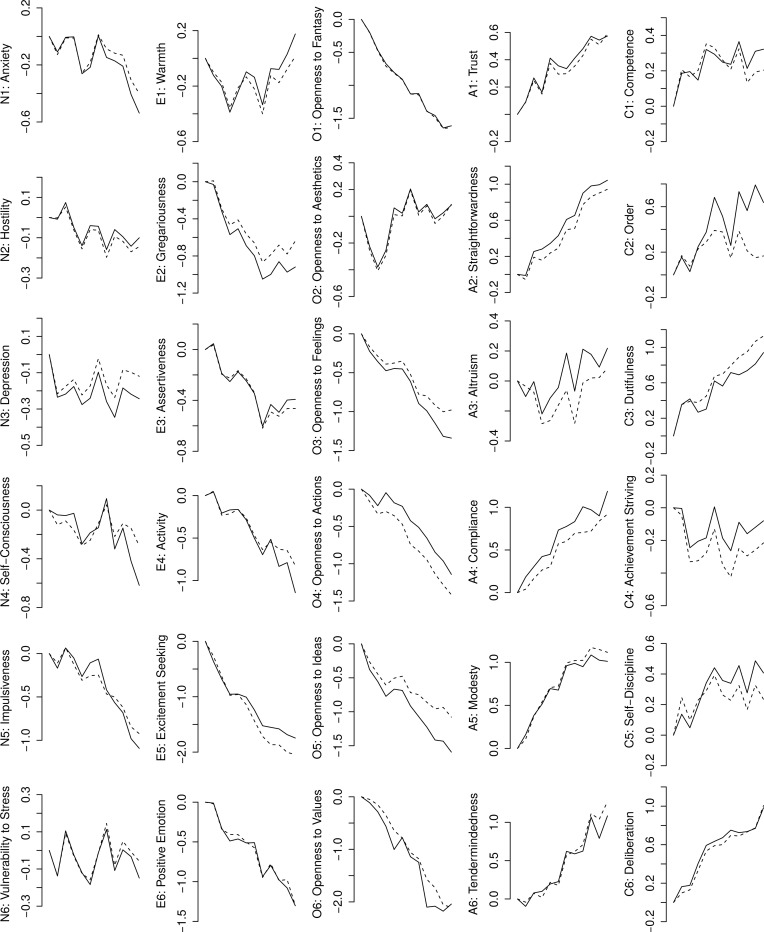
Age differences in the 30 NEO-PI-3 facets before (dashed lines) and and after (solid lines) establishing partial measurement invariance. Horizontal axis represents 12 age groups. Vertical axis represents the mean age group scores (in the standard deviation units of the youngest group).

To test whether allowing some loadings and intercepts to vary freely across age groups had any impact on age group differences, we compared mean facet scores before (i.e., based on the poorly fitting models with loadings and intercepts set equal in all groups) and after establishing partial invariance. Both types of means for all facets are shown in Fig. 2. In most facets, the differences appeared to be rather small, but there were some exceptions. For example, in the C2: Order, mean scores from the model with all loadings and intercepts constrained equal tended to show curvilinear associations with age, whereas in the partial invariance model the scores tended to increase throughout life. Or, the scores in N4: Self-Consciousness or O5: Openness to Ideas tended to decline faster in older age based on partial invariance models compared to the models with all loadings and intercepts constrained equal.

These results seem to indicate that in most facets the lack of MI had no considerable practical implications for the interpretation age group differences. Why? The most likely reason for this was that the items that caused the lack of MI in the first place diverged from the typical age-trends of their respective facets in both directions (e.g., had both weaker or stronger age-effects than the main age-trajectory to the respective facet); some evidence for this is presented below by addressing within-facet variability in how item residual variances correlated with age. If so, their unique age-trajectories cancelled each other out in the full model but contributed to its poor fit, and removal of their equality constraints simply reduced the misfit.

#### Implications of partial MI

Although establishing partial MI did not make much of a difference to the observed age-differences in scale scores, two things need to be noted. First, to the extent that a number of measurement model parameters were allowed to vary across age groups (especially intercepts), the partially invariant latent traits were defined differently at different ages and therefore the trait-scores did not reflect exactly the same constructs in different age groups. However, the second and potentially even bigger concern is that scale scores simply appeared to mask possibly important age-differences at the level of single items. We illustrate this situation in the next section.

### The Meaning of the Lack of Strong MI

#### Age group differences in the unique variance in items

The cause of the lack of strong MI was that age differences in single items were different from those in the facets the items were designed to measure. To obtain some idea of the qualitative importance of this, we obtained FSs (calculated as described above) for each facet and residualized all 240 items for their respective Fss. These residuals represented variance in individual items that was independent of the variance that all items of the same facet shared (i.e., variance independent of the ostensible trait). We then calculated linear correlations of these residual variances with age, considering significant only correlations r >. 071 (we adjusted significance threshold for multiple tests, based on the number of test items: p = .05 / 240 = .0002). These analyses were carried out in the full sample.

The residuals of 110 items (45.8% of all items) had significant (p <. 0002) linear associations with age (164 items had such correlations without being residualized). These 110 items were distributed across 29 of the 30 facets, with most facets having three (8 facets), four (5 facets) or five (6 facets) items with significant residual age-correlations, but some facets having six or more (in O1: Openness to Fantasy, only one item had significant residual age-correlation). In 28 of the 29 facets, some items had significant negative and some significant positive residual correlations with age. This is in line with the explanation above for the relatively small effect of establishing partial MI on the average scale scores of age groups. The average significant residual age-correlation was r = |0.13| (SD = 0.06, maximum 0.39). Given that average age-correlation in these 110 items *before* residualizing was |0.15| (SD = 0.10, maximum 0.45), the latent facets these items were intended to measure could not account for substantial amounts of their age-related variance.

#### Examples of the lack of MI

**Fig 3:**
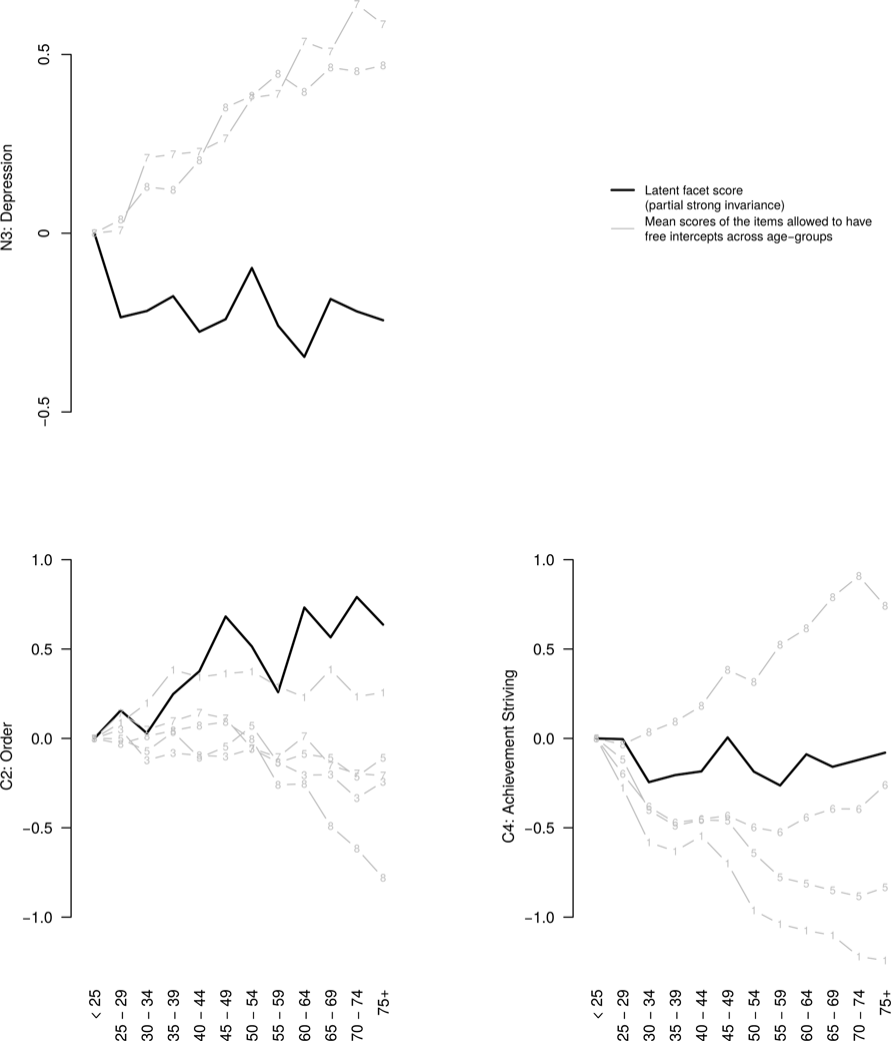
Latent trait scores of three facets and scores of single items that had their intercept equality constraints relaxed to obtain partial strong measurement invariance. Horizontal axis represents 12 age groups. Vertical axis represents the mean age group scores (in the standard deviation units of the youngest group).

To further illustrate the meaning of the lack of strong MI, three examples are depicted in Fig. 3. The N3: Depression scores (from the partial strong invariance model) showed a slight downwards trend with age. However, two items had significant linear positive associations with age (item #5: “I tend to blame myself when anything goes wrong”; r = .17; item #6: “I have a low opinion of myself”; r = .20). Therefore, whereas older people tended to feel slightly less negative emotions than younger people (based on the common variance), they were inclined to be more self-critical, given their levels in N3: Depression. This finding may make sense as it may be a sign of maturation to allow for self-criticism and yet not feel excessively bad about this. This divergence among the items of the facet might be of great theoretical importance for understanding personality maturation and age (or cohort) differences in manifestation of depression.

While older people tended to score increasingly higher on the C2: Dutifulness facets than younger people after establishing partial strong MI, this tendency, in fact, applied only to three of its eight items. Five items showed different developmental patterns. The item (#8) that most clearly opposed the facet-level trend was: “I spend a lot of time looking for things I’ve misplaced” (reverse-scored). The clear downward trend (reflecting greater time spent on looking for lost things) for this item after middle-age was consistent with the general trend of memory/cognitive decline [60] and it may be of theoretical interest for understanding age differences in orderliness: while the items that referred to a *preference* for keeping things neat and organized and being fastidious were positively associated with age, the downward trend for this item indicated that older people may be increasingly less successful in *being* organized. Note that this facets was also one of the most notable ones to fail the weak MI test, suggesting invariance in factor loadings.

There were no clear age differences in self-reported C4: Achievement Striving scores. However, some items showed substantial age-related differences (up to 1.2 standard deviations). Scores on item #8 (“I’m something of a “workaholic””) increased until age 70 and then showed slight decline (possibly partly reflecting physical limitations to work or retirement) and scores on two reverse-scored items #1 (“I am easy-going and lackadaisical”) and #5 (“I don’t feel like I’m driven to get ahead”) showed decreases; the difference between item #1 and item #8 was 2.1 standard deviations around age 70 (while the scores were identical in the youngest age group). Therefore, although older people (or cohorts) may describe themselves as more hard-working than young people do, they may have less ambitions and feel less driven to get ahead, for example, due to being already retired or approaching retirement.

Of note is that in many other cases, the patterns of positive *vs* negative residual age correlations were not so readily interpretable.

### Age Group Differences in the Facet Scores

**Fig 4:**
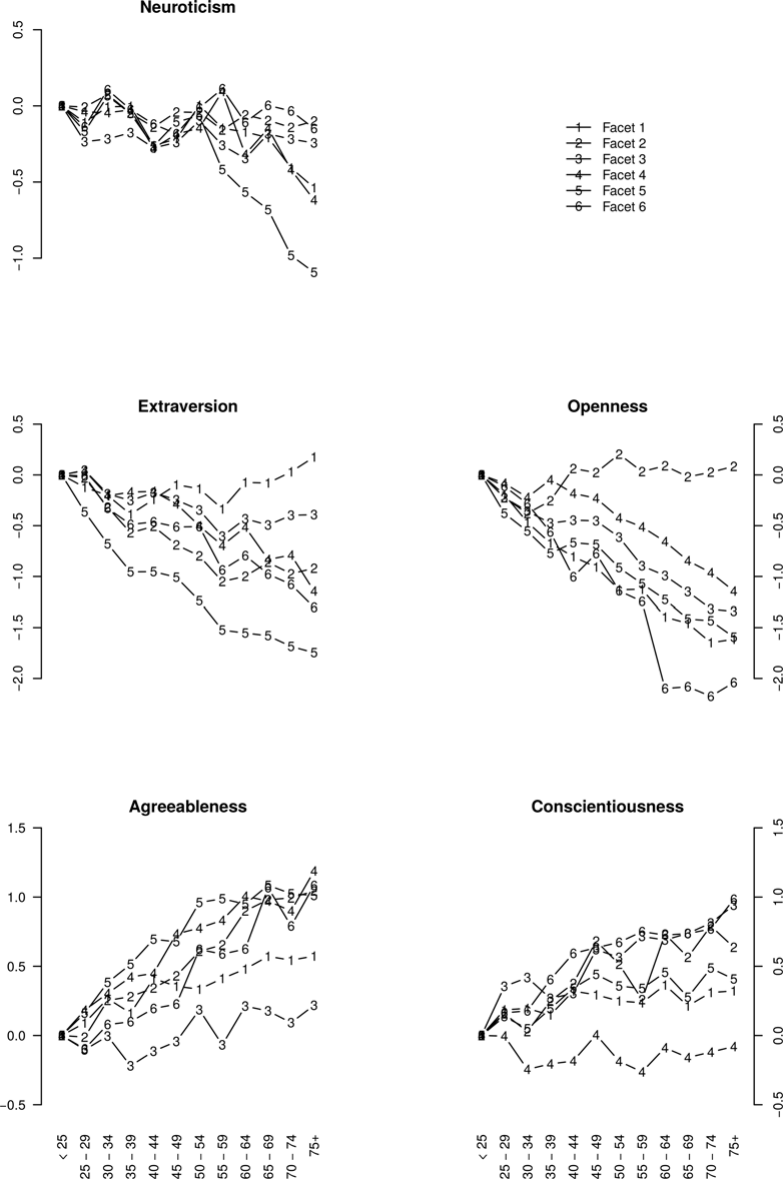
Age differences in NEO-PI-3 facet scores. The scores are based on measurement models that demonstrated partial measurement invariance. The facets are number according to the NEO-PI-3 manual (see also Table 2). Horizontal axis represents 12 age groups. Vertical axis represents the mean age group scores (in the standard deviation units of the youngest group).

We now move from the level of items to the level of facets. Fig. 4 depicts age differences in the 30 facets after having established partial MI. As the facets are grouped according to their FFM domains (unlike in Fig. 2, which focuses on the implications of partial MI), it is easy to see that sometimes the facets of the same FFM domain displayed notably distinct age differences.

In the Neuroticism domain N2: Hostility, N3: Depression and N6: Vulnerability showed little age differences, whereas scores in N1: Anxiety, N4: Self-Consciousness and, especially, N5: Impulsiveness were lower in older people than in younger and middle-aged people. In the Extraversion domain, there were curvilinear age differences in E1: Warmth (upwards in older aged people) and in E2: Gregariousness and to a lesser extent in E3: Assertiveness (drop in middle-aged and older people compared to younger people), whereas E5: Excitement-Seeking and E6: Positive Emotions scores, and to a less extent E4: Activity scores, were all lower in older than in younger ages, although to different degrees. The most deviant facet, E1: Warmth, was also one of the two facets failing to show even configural MI (which could not be patched with partial MI). In the Openness domain, scores on O2: Openness to Aesthetics were relatively similar in all age groups, whereas scores on other facets tended to be lower in older age groups, although to varying degrees. The scores on O6: Openness to Values appeared to be especially low in people in their late fifties and older. However, O6: Openness to Values was the facet that most clearly failed to show even configural MI. In the Agreeableness domain, age had a negligible positive association with A3: Altruism, somewhat stronger positive association with A1: Trust but stronger positive correlations with the other four facets. In the Conscientiousness domain, age differences in C4: Achievement Striving were negligible. C1: Competence and C5: Self-Discipline had somewhat higher scores in people in their late thirties and older than in younger people. Levels of C2: Order, C3: Dutifulness, and C6: Deliberation were increasingly higher in older age groups (although the trajectory was very wriggly for C2: Order).

Overall, there appeared to be notable variability among the facets of the same domains, just as there had been variability in age-trajectories among the items of those facets.

### Variability among Facets in Age Group Differences: Formal Tests Using the MI Framework

To test whether the age trajectory differences among facets that were described in the previous section reflected more than random flukes or variability among facets in factor loadings, we relied again on the MI framework. Specifically, lack of strong MI would indicate that differences among facets in age trajectories were significant.

#### Establishing baseline models

Before testing for MI using multi-group CFA, we first constructed single-group CFA models for each of the five FFM domains, with domains defined by the FSs of their respective facets (obtained as described above). All of these models needed two or more correlated residual variances to fit data acceptably (see Table 3; all models with standardized loadings and residual correlations are given in S2 File). The fit indices of all single-group and multi-group models are given in S3 File.

**Table 3:** Fit statistics for the models of the FFM domains.

	No residual variance correlations			With residual variance correlations			
	χ2	CFI	RMSEA [90% CI]	χ2	df	CFI	RMSEA [90% CI]
*Self-Ratings*						
Neuroticism	377.79	.933	.123[.113;. 133]	59.69	6	.990	.058[.045;. 070]
Extraversion	620.36	.884	.158[.149;. 169]	32.36	6	.995	.040[.028;. 054]
Openness	659.22	.794	.163[.154;. 173]	87.94	6	.974	.071[.059;. 083]
Agreeableness	717.56	.697	.171[.160;. 181]	70.55	4	.972	.078[.064;. 094]
Conscientiousness	285.24	.941	.107[.097;. 116]	105.61	7	.979	.072[.062;. 083]

NOTE: χ2 = Chi-square; df = degrees of freedom; CFI = Comparative Fit Index; RMSEA = Root Mean Square Error Approximation; CI = confidence intervals. * Degrees of freedom = 9. The number of the pair of residual variance correlations equals 9 minus df (column 6). Chi-square statistics were significant at p <. 001 in all cases.

#### MI

All domains showed configural MI according to the ΔCFI and RMSEACI criteria. Also, weak MI held for all domains except Agreeableness (ΔCFI = 0.02; RMSEA confidence intervals overlapped). However, no FFM domain reached strong MI, with ΔCFI = .02,. 12,. 32,. 06, and. 08, respectively for Neuroticism, Extraversion, Openness, Agreeableness, and Conscientiousness. Also, Extraversion, Openness, and Conscientiousness did not reach strong MI according to the RMSEACI criterion. Openness, Agreeableness and Conscientiousness did not reach strict MI according to the ΔCFI criterion (ΔCFI. 02 to. 03). In sum, the MI tests confirmed that variability among the facets in age group differences was beyond that expected by chance or differential factor loadings of the facets on their latent domain factors. In other words, facets of the same domains varied significantly in terms of their age trajectories.

#### Partial MI

In order to establish partial weak MI (ΔCFI ≤. 01), the equality constraints on the factor loadings of self-reported A4: Compliance and A5: Modesty on the latent Agreeableness factors had to be released. To establish partial strong MI for Neuroticism, only one intercept equality constraint (for N5: Impulsiveness) had to be relaxed. In other traits, however, more intercepts had to be released. For Extraversion, the intercept constraints of E1: Warmth, E5: Excitement-Seeking and E6: Positive Emotions had to be relaxed. For Openness, intercepts of O2: Openness to Aesthetics, O3: Openness to Emotions, O5: Openness to Ideas and O6: Openness to Values were released. For Agreeableness, the intercept equality constraints were released for A1: Trust and A3: Altruism. For Conscientiousness, intercepts were released for C3: Dutifulness, C4: Achievement Striving and C6: Deliberation. Overall, 13 of the 30 intercepts were released. In other words, about 43% of the facets varied across age groups differently from the common variance each of them shared with the other facets of the same FFM domain. Apparently, thus, inconsistent age group differences across the facets of the same domains were common.

### Age Group Differences that the FFM Domains Captured or Ignored

#### Age trends before and after MI

**Fig 5:**
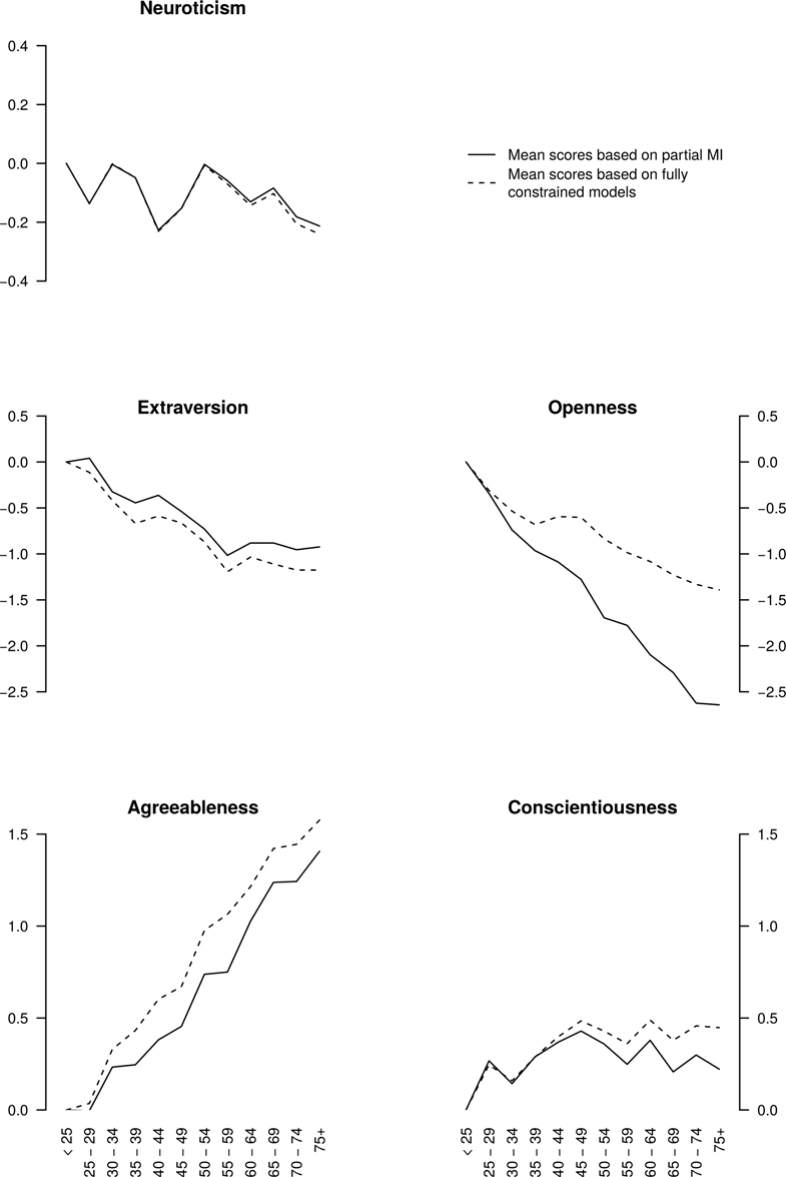
Age differences in FFM domain scores before and after establishing partial measurement invariance. Horizontal axis represents 12 age groups. Vertical axis represents the mean age group scores (in the standard deviation units of the youngest group).

Fig. 5 depicts mean-level differences across the 12 age groups in the FFM domain scores both before and after establishing partial strong MI. There was little systematic age-related variability in the Neuroticism scores, with the two types of mean scores yielding similar conclusions. Extraversion had progressively lower scores in older age groups, with the age differences being slightly less pronounced after establishing partial MI; this was because the contribution of E5: Excitement Seeking that had shown the strongest age group differences was set free. In the Openness domain, mean scores differed notably depending on whether partial MI was established or not: the linear downwards trend was substantially stronger after establishing partial MI. In the oldest group the mean Openness score was 1.25 standard deviations lower in the partial MI-based results than in the results based on measurement models that were identical across the age groups; this was because of the removal of the fixed contribution of O2: Openness to Aesthetics. In Agreeableness, the results depended less on whether partial MI was established or not; there was a fairly linear trend for older people to score higher than younger people. For Conscientiousness, the scores were the highest in middle-age based on the partially invariant measurement models but stayed at the same level from middle-age to older age based on measurement models that were identical across age groups. Therefore, allowing or not allowing for partial MI influenced the conclusions to some extent, especially for Openness.

#### Domain-level findings masked information

Obviously, these mean-level differences in domain scores masked the variability among facets in their age group differences (see Fig. 4) and establishing partial MI did not fix but only helped to hide this issue. To illustrate this, we residualized the FSs of the facets for the FSs of their respective domains and calculated linear correlations of these residuals with age. The domain FSs were obtained from ordinary least squares factor analyses based on the FSs of their respective facets.

In this paragraph, every correlation of |0.05| or higher was significant at p < 0.01, whereas facet-age correlations above 0.06 where significant at a Bonferroni-corrected p-value of p = 0.05/30 = 0.0017. While the Neuroticism domain scores correlated with age at r = -0.05, most of its facets had notably stronger associations with age after being residualized for the domain: r = 0.09 (N2: Angry Hostility), -0.39 (N3: Depression), -0.07 (N4: Self-Consciousness), -0.20 (N5: Impulsiveness), and -0.08 (N6: Vulnerability). Extraversion domain scores had a stronger negative association with age (r = -0.32), but some of its facets also had significant residual age correlations: r = 0.29 (E1: Warmth), -0.10 (E3: Assertiveness), 0.16 (E4: Activity), and 0.25 (E6: Positive Emotions). Openness scores had a negative association with age (r = -0.41), but several of its facets also had significant residual age-correlations: r = 0.07 (O1: Openness to Fantasy), -0.17 (O3: Openness to Feelings), -0.29 (O4: Openness to Actions), 0.16 (O5: Openness to Ideas) and -0.25 (O6: Openness to Values). Agreeableness factor scores had a positive correlation with age (r = 0.35), whereas three of its facets had significant residual age-correlations: r = 0.09 (A2: Straightforwardness), 0.36 (A3: Altruism), and -0.08 (A6: Tendermindedness). Conscientiousness had a positive correlation with age (r = 0.11) and five of its six facets had significant residual age-correlations: r = -0.19 (C1: Competence), 0.08 (C2: Order), 0.05 (C4: Achievement Striving), -0.07 (C5: Self-Discipline), and 0.22 (C6: Deliberation).

These findings, quantifying the amount of residual age-related variance in facets, again, showed that facets of the same purported FFM domains varied systematically in terms of age group differences. Sometimes the unique variances of the facets of the same domains had opposing age group differences. Therefore, studying age group differences only at the level of the FFM domains is likely to result in a lot of age-related trends remaining uncovered.

#### Sensitivity Analyses for the FFM-Age Associations

Personality psychologists often assume that the items of a latent FFM domain are its exchangeable (i.e., random) indicators that contain both domain-related and unique variance, including systematic variance that is unique to particular facets. If so, one way of testing whether the relationship of age with the latent domain scores as such is robust or sensitive to particular sets of its indicators is to define the domain scores via items that are randomly drawn from among all items designed to measure the domain, regardless of their intended facet. Presumably, then, any facet-specific or otherwise unique variance will be suppressed in such ‘random’ scales, leaving domain-related (i.e., pure FFM) variance to dominate. In a number of such ‘random’ latent trait scores, their correlations with age will provide a test of robustness of the domain-age association.

Based on such reasoning, we created 1,000 ‘random’ latent traits for each of the FFM domains and calculated their correlations with age; the ranges of 95% of these correlations (i.e., the 2.5% and 97.5% quantiles) are reported along with the mean. Each ‘random’ latent trait represented a factor score of 12 (25%) items randomly chosen from among the 48 items of the given domain. In the Neuroticism domain, the average correlation of the 1,000 ‘random’ latent traits with age was-0.04, and the 95% range varied from-0.11 to 0.03. In the Extraversion domain, the average correlation with age was-0.29 and the 95% range varied from -0.41 to -0.17. In the Openness domain, the average correlation with age was-0.30, and the 95% range varied from -0.46 to -0.03. In the Agreeableness domain, the average correlation with age was 0.29, and the 95% range varied from 0.11 to 0.41. Finally, in the Conscientiousness domain, the average correlation with age was 0.09, and the 95% range varied from-0.04 to 0.22.

Thus, there was notable variability in how the FFM domains were associated with age, depending on the particular random quarter of indicators by which they were defined. In other words, the conclusions regarding age group differences were sensitive to the operationalization of the construct. This again suggests that conceptualizations of the FFM domains may not be aetiologically consistent and age differences in personality could be better described using more specific trait constructs.

## Discussion

Science strives to discover general laws that can provide parsimonious predictions and explanations for relevant observable phenomena. However, the general laws are useful only to the extent that they allow for reasonably accurate predictions and explanations. Much of current personality psychology, focusing mainly on differences among people, is based on the general theoretical principle that individual differences in a observable behaviours, thoughts and feelings tend to co-vary along five dimensions, the FFM domains [61], pointing to five presumably coherent mechanisms (latent traits) that *cause* these observable individual differences [13,16]. Metaphorically speaking, there are five currently unobservable but presumably somehow biologically rooted (here in the aetiological sense, as ultimately all behaviour is caused by brain processes) ‘generators’ within each individual that cause the observable manifestations of personality traits. Individual differences in these manifestations result from differences in the power of these ‘generators’: some people have more powerful, say, ‘extraversion generator’ than others and therefore they display more behaviour of the Extraversion kind (in interaction with other traits and environment or unique genes/biology).

If this theoretical premise is correct, it would seem to follow that age group differences in people’s behaviours, thoughts and feelings should be largely caused by differences in these generators and therefore adhere to the five-dimension organisation, so that, for example, the definition of Extraversion is the same in people of age 60 as it is in people of age 50. Thereby, the indicators of Extraversion should exhibit similar factor loadings and age-differences, here operationalized as strong MI. In fact, establishing at least strong MI is an essential requirement if we are to compare mean levels of Extraversion across the age groups. If it does not hold, we are essentially comparing qualitatively different phenomena. Of course, it is possible to claim that any failure to meet this MI requirement can be ascribed to sources of variance that are independent of the five generators; we will address this possibility below.

Based on some previous studies as well as the present results, the MI requirement may often be met to only a limited degree as the facets of the purported FFM domains show rather different patterns of age differences. Furthermore, in our analyses the items comprising the facets themselves tended to vary inconsistently with age: different items of the same facets often showed different, sometimes even opposing, relations with age. These findings have major implications for understanding and describing personality development and perhaps personality structure more generally. We understand that the structure and development of personality are inherently interwoven and cannot be understood separately. If one wants to describe the development of traits, one will have to define the traits in the first place. Likewise, the definition of a unitary trait calls for consistency in its development [14].

### Implications for Personality Conceptualizations

The inability to establish at least strong measurement invariance casts doubt on the FFM domains and facets being coherent trait conceptualizations, at least as embodied in the NEO-PI-3 questionnaire. In particular, if the facets of the same domains and items of the same facets display substantially varying age group differences, it is possible that they do not share the same underlying proclivity or aetiology. For example, two age-trajectories may suggest (at least) two (at least partially distinct) developmental mechanisms.

This raises an obvious question: if there is evidence that indicators of a trait may not share a common underlying aetiology, how do we know that a common trait was there in the first place? Two aetiologies may suggest two traits rather than one. This is very similar to the problem faced in cognitive abilities research: apparently robust general cognitive ability seems to have aspects that display substantially different developmental trajectories, suggesting that the construct is not aetiologically homogeneous. While some researches simply acknowledge that theories of intelligence need to account for this developmental decoupling [18], others suggest that the decoupling points to the need to consider intelligence as having multiple dimensions rather than one [24]. Clearly, the developmental inconsistency cannot be ignored.

Indeed, covariance of trait indicators at any given time-point does not, in itself, speak for the existence of a common latent proclivity. The covariances may occur just as naturally through emergence processes rooted completely in externally influenced experiences or due to causal interconnections among the indicators [59,62]. Emergence-based explanations for cross-sectional correlations do not require that the indicators of the purported traits display similar group differences. Furthermore, such emergence-based explanations for trait-indicator intercorrelations are consistent with our finding that unidimensional latent trait models tended to fit poorly, with an average of two item pairs per facet (see Table 2) and tens or hundreds of item pairs per domain having correlated residual variances (i.e., items co-varyinig over and above their shared variance with other items of the same facet or domain). That is, one of the reasons for substantial residual co-variances might be that latent trait models were not optimal representations of the associations present in data, as the purported unobservable traits did not appear to be able to account for them. Taken together, the variability among trait-indicators in age group differences combined with poor fit of unidimensional measurement models is not consistent with there being coherent FFM domains and facets.

Our findings may suggest that researchers interested in coherent trait conceptualizations with unitary aetiology may want to look beyond the FFM domains. Although one could, then, think of describing individual differences in personality traits using the facets, which have been shown to demonstrate both unique underpinnings [63] and relations with external variables [64], the present findings show that facets, at least as implemented in the NEO-PI-3, suffer from the same problems as the FFM domains, as their items tend to vary substantially in age group differences. As a result, alternative trait models may also be considered. Which exactly these models may be is a matter of future research. Most likely these models would postulate a larger number of specific but “tight”, narrowly defined trait units rather than a small number of overarching but “wobbly” traits. That considering specific personality characteristics, perhaps reflected in single items, may prove valuable was also recently pointed out by McCrae [13]; he called such specific characteristics ‘nuances’.

Taxonomies of the nuances, however, remain to be created. One approach to developing them would be analysing more closely the residual co-variances between items of the same facets such as those specified in the present study. These residual co-variances may reflect trivial redundancies between items (two items only slightly differing in wording) but they may also point to nuances [13]. To the extent that the non-trivial residual co-variances could prove replicable across datasets, preferably sampling individuals from different demographic backgrounds and cultures, more or less universal taxonomies could be established.

### Implications for Studying Personality Development

If the present findings suggest that the FFM domains and facets, at least as operationalized in the NEO-PI-3, may not provide a sufficiently coherent account of personality differences, this inevitably suggests that the usefulness of these traits for describing and understanding personality *development* may also be somewhat limited. Based on the present findings, describing age group differences at the level of the FFM domains entails a substantial loss of information, given the variability in the age group differences among facets. Likewise, describing age group differences at the level of facets ignores the variability in item correlations with age. But even more importantly, studying personality development in units that may have multiple, possibly somewhat independent underlying mechanisms may be simply inefficient and possibly misleading. Relatedly, studies looking for molecular genetic bases of the FFM traits (i.e., their biological roots) may have been relatively unsuccessful due to the domains and facets having multiple more or less independent underlying mechanisms with different genetic architectures [65]. On a related note, it has been show that broad FFM traits may provide suboptimal prediction of outcomes, because of substantial heterogeneity among their constituents in outcome-correlations [66].

### The Unique Variance Problem

Of course, one might argue that the variability in age group differences among the facets of the FFM domains pertains to the unique variance in the facets, as facets may reflect additional sources of variance in addition to those caused by the domains (defined as the common variance) that they are supposed to articulate [63]. The same can also be true for variability among items of the same facets in terms of age group differences. Indeed, this is a plausible explanation. However, two problems remain.

First, this possibility is hard to test, given that the FFM domains and facets cannot be defined and operationalized independently of the items, at least currently. However carefully crafted, domain and facet scores remain composites (or, in the best case, common variance) of human-made personality test items rather than tangible, linearly ordered attributes of human brains. Moreover, what is common variance depends on the particular personality measures and samples that are used to define it, as is also evidenced by relatively moderate correlations between scales measuring the same purported traits [67]. Of course, in any given study, it is possible to partition variance in items into the variance they share with other items of the same trait (be it facet or domain) and unique variance, and then link these variances separately to age; we did this in the present study. But is the common variance a valid reflection of a latent trait (the underlying ‘generators’) that generalizes across measures and samples and is its association with age therefore a reasonable estimate of the effect of age on the latent trait? Quite clearly, the less coherent the indicators of the latent trait are both cross-sectionally and across development, the less tenable this hypothesis becomes.

We attempted to test the extent to which the age group differences were robust across particular operationalizations of the latent traits. For this, we created a series of latent trait scores for each of the FFM domains using random quarters of its 48 items. It seems a sensible assumption that latent trait scores from *random* samples of indicators of a domain allow for the most natural operationalizations of the trait, as such scores should suppress any systematic but domain-irrelevant (e.g., facet-specific) variance and let only the domain-related variance shine through. For all of the FFM domains, their correlations with age varied notably depending on which random quarter of their items was used to define them. This goes to show that the common variance of items is a far from perfect opeationalization of latent traits, at least when it comes to investigating age group differences of personality traits.

The second problem with arguing that it may be only the unique variance of trait-indicators that causes the diversity in age group differences rather than the latent traits as such is more general. In particular, it illustrates the kind of reasoning that makes many theoretical positions regarding personality traits unfalsifiable. The reasoning may go as follows. If empirical data do not support a postulated measurement model, as was the case in the present study, one can argue that personality is simply too complex to adhere to a well-defined theoretical trait model and dismiss the misfit [47,32,68], although not everyone agrees with this position [69,70]. In this view, there can be all sorts of unknown but apparently legitimate sources of variance and co-variance in addition to those specified in the model. Next, since there is no measurement model that allows for clearly separating the true (but unobserved) traits from other (again unobserved, facet- or item-specific) sources of variance, any ‘unexpected’ result—such as age group differences in facets or items that do not match the traits they are supposed to define—can be explained away by blaming it on the ‘unknown’ sources of variance.

This may surely be true, but it is nearly impossible to empirically verify, given the current status of personality traits as *unobserved* attributes of human mind. We think that this is a slippery road. Since latent personality traits are by their very nature unobserved, extra care needs to be taken that any indirect evidence in favour of and in relation to them is as solid as possible. A possible solution might be giving up on broad and incoherent trait conceptualizations and focusing on traits that can be better defined and measured, and that stick together over the lifespan.

### Limitations

From the age differences point of view, a limitation of our study was that it was entirely cross-sectional and therefore the observed age group differences might have reflected cohort differences in addition to, or instead of, age-differences *per se*. For example, the historical transitions in Estonia may have influenced some age groups differently than others. Furthermore, there may have been sampling bias differences across age levels. This does not, however, undermine our discussion regarding the implications of the findings for traits more generally. Even if the age group differences reflect only cohort-differences, for example, the finding that facets and items of the same traits show variable cohort differences is not consistent with them being manifestations of coherent traits.

As another limitation, although the total sample was relatively large, the single age groups were not. As a result, the MI testing may have been somewhat underpowered and the lack of MI may have been underestimated.

The study was conducted in a specific country with its particular social and historical characteristics and language. Although currently there is no evidence that these counry-specific factors drove the inconsistencies in the cross-sectional age group differences of the manifestations of the same ostensible traits, this remains plausible. The findings should therefore be replicated elsewhere.

### Conclusions

In sum, we observed that the measurement models of the FFM domains and their facets that often fitted empirical data somewhat poorly from the outset could not be defined in the same way across adulthood as facets of the same domain and items of the same facets varied considerably in their age group differences. This is not consistent with the domains and facets being coherent trait conceptualizations that could serve well for describing and understanding personality development. This, in turn, suggests that personality psychology may benefit from considering alternative and more coherent units of analyses.

## Supporting Information

S1 File Data. Means, standard deviations, numbers of observations and co-variance matrices for all age groups.
(ZIP)

S2 File Measurement Models. Standardized measurement model estimates for the NEO Personality Inventory 3 (NEO PI-3) facets (N1 … C6, see Table 2 of the main text for full facet names) and domains (N = Neuroticism, E = Extraversion, O = Openness to Values, A = Agreeableness, C = Conscientiousness).
(PDF)

S3 File Reliabilities and fit indices. Reliabilities of facet- and domain-level latent traits across age groups and fit indices for single-group and multi-group models.
(ODS)
