# Supplementary material for: Within-Trait Heterogeneity in Age Group Differences in Personality Domains and Facets: Implications for the Development and Coherence of Personality Traits
Source: PLoS One. 2015 Mar 9;10(3):e0119667. doi: 10.1371/journal.pone.0119667 (PMC4353719; doi:10.1371/journal.pone.0119667)
Supplement: S2 File — Standardized measurement model estimates for the NEO Personality Inventory 3 (NEO PI-3) facets (N1 … C6, see Table 2 of the main text for full facet names) and domains (N = Neuroticism, E = Extraversion, O = Openness to Values, A = Agreeableness, C = Conscientiousness). (PDF) [file pone.0119667.s002.pdf]

Supporting Information S1. Measurement Models.

Standardized measurement model estimates for the NEO Personality Inventory 3 (NEO PI-3) facets (N1 … C6, see Table 2 of the main text for full facet names) and domains (N = Neuroticism, E = Extraversion, O = Openness to Values, A = Agreeableness, C = Conscientiousness).

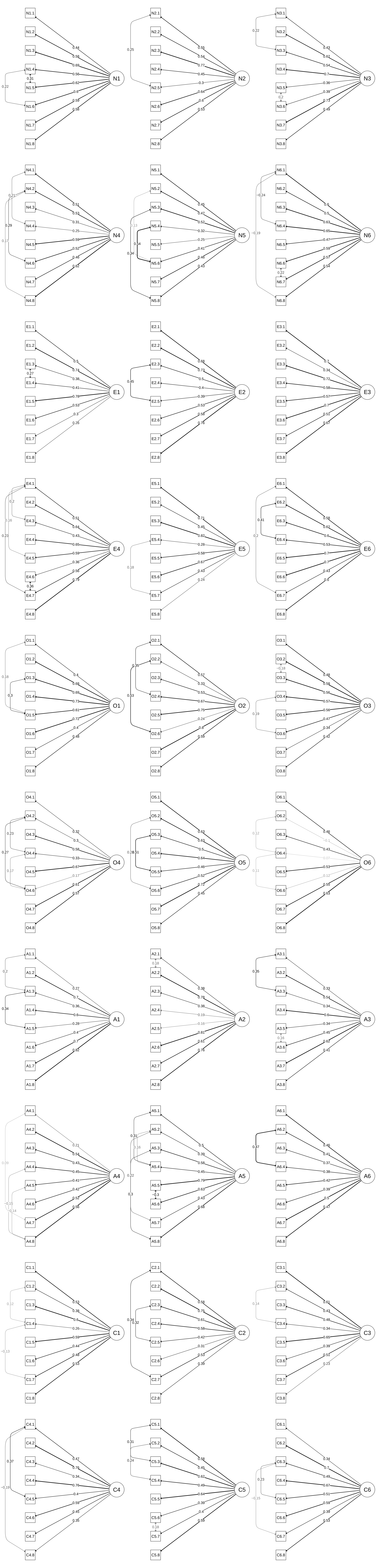

N1.1 N1.2 N1.3 N1.4 N1.5 N1.6 N1.7 N1.8 N1
N2.1 N2.2 N2.3 N2.4 N2.5 N2.6 N2.7 N2.8 N2
N3.1 N3.2 N3.3 N3.4 N3.5 N3.6 N3.7 N3.8 N3
N4.1 N4.2 N4.3 N4.4 N4.5 N4.6 N4.7 N4.8 N4
N5.1 N5.2 N5.3 N5.4 N5.5 N5.6 N5.7 N5.8 N5
N6.1 N6.2 N6.3 N6.4 N6.5 N6.6 N6.7 N6.8 N6
E1.1 E1.2 E1.3 E1.4 E1.5 E1.6 E1.7 E1.8 E1
E2.1 E2.2 E2.3 E2.4 E2.5 E2.6 E2.7 E2.8 E2
E3.1 E3.2 E3.3 E3.4 E3.5 E3.6 E3.7 E3.8 E3
E4.1 E4.2 E4.3 E4.4 E4.5 E4.6 E4.7 E4.8 E4
E5.1 E5.2 E5.3 E5.4 E5.5 E5.6 E5.7 E5.8 E5
E6.1 E6.2 E6.3 E6.4 E6.5 E6.6 E6.7 E6.8 E6
O1.1 O1.2 O1.3 O1.4 O1.5 O1.6 O1.7 O1.8 O1
O2.1 O2.2 O2.3 O2.4 O2.5 O2.6 O2.7 O2.8 O2
O3.1 O3.2 O3.3 O3.4 O3.5 O3.6 O3.7 O3.8 O3
O4.1 O4.2 O4.3 O4.4 O4.5 O4.6 O4.7 O4.8 O4
O5.1 O5.2 O5.3 O5.4 O5.5 O5.6 O5.7 O5.8 O5
O6.1 O6.2 O6.3 O6.4 O6.5 O6.6 O6.7 O6.8 O6
A1.1 A1.2 A1.3 A1.4 A1.5 A1.6 A1.7 A1.8 A1
A2.1 A2.2 A2.3 A2.4 A2.5 A2.6 A2.7 A2.8 A2
A3.1 A3.2 A3.3 A3.4 A3.5 A3.6 A3.7 A3.8 A3
A4.1 A4.2 A4.3 A4.4 A4.5 A4.6 A4.7 A4.8 A4
A5.1 A5.2 A5.3 A5.4 A5.5 A5.6 A5.7 A5.8 A5
A6.1 A6.2 A6.3 A6.4 A6.5 A6.6 A6.7 A6.8 A6
C1.1 C1.2 C1.3 C1.4 C1.5 C1.6 C1.7 C1.8 C1
C2.1 C2.2 C2.3 C2.4 C2.5 C2.6 C2.7 C2.8 C2
C3.1 C3.2 C3.3 C3.4 C3.5 C3.6 C3.7 C3.8 C3
C4.1 C4.2 C4.3 C4.4 C4.5 C4.6 C4.7 C4.8 C4
C5.1 C5.2 C5.3 C5.4 C5.5 C5.6 C5.7 C5.8 C5
C6.1 C6.2 C6.3 C6.4 C6.5 C6.6 C6.7 C6.8 C6

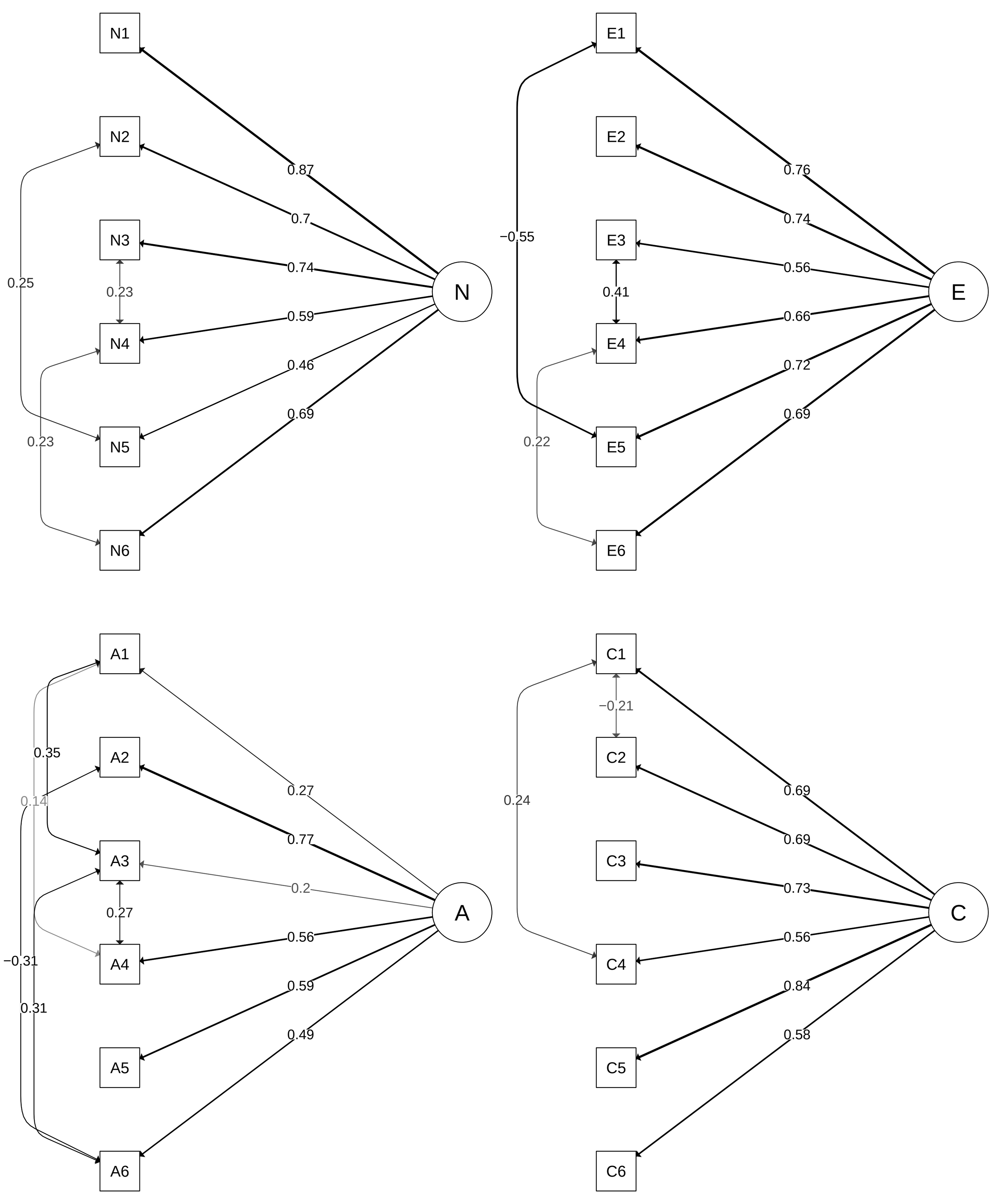
N1
N2
N3
N4
N5
N6
N
0.87
0.7
0.74
0.59
0.46
0.69
0.25
0.23
0.23
E1
E2
E3
E4
E5
E6
E
0.76
0.74
0.56
0.66
0.72
0.69
−0.55
0.41
0.22
O1
O2
O3
O4
O5
O6
O
0.59
0.54
0.72
0.48
0.82
0.32
0.2
−0.62
0.43
A1
A2
A3
A4
A5
A6
A
0.27
0.77
0.2
0.56
0.59
0.49
0.35
0.14
0.27
−0.31
0.31
C1
C2
C3
C4
C5
C6
C
0.69
0.69
0.73
0.56
0.84
0.58
−0.21
0.24
